# Development and Validation of a Nonremission Risk Prediction Model in  First-Episode Psychosis: An Analysis of 2 Longitudinal Studies

**DOI:** 10.1093/schizbullopen/sgab041

**Published:** 2021-08-31

**Authors:** Samuel P Leighton, Rajeev Krishnadas, Rachel Upthegrove, Steven Marwaha, Ewout W Steyerberg, Georgios V Gkoutos, Matthew R Broome, Peter F Liddle, Linda Everard, Swaran P Singh, Nicholas Freemantle, David Fowler, Peter B Jones, Vimal Sharma, Robin Murray, Til Wykes, Richard J Drake, Iain Buchan, Simon Rogers, Jonathan Cavanagh, Shon W Lewis, Max Birchwood, Pavan K Mallikarjun

**Affiliations:** Institute of Health and Wellbeing, University of Glasgow, Glasgow, UK; Institute of Neuroscience and Psychology, University of Glasgow, Glasgow, UK; Institute for Mental Health, University of Birmingham, Birmingham, UK; Institute for Mental Health, University of Birmingham, Birmingham, UK; Faculty of Medicine, University of Leiden, Leiden, Netherlands; Institute of Cancer and Genomic Sciences, University of Birmingham, Birmingham, UK; NIHR Surgical Reconstruction and Microbiology Research Centre, Birmingham, UK; NIHR Biomedical Research Centre, Birmingham, UK; MRC Health Data Research UK (HDR), Midlands Site, UK; Institute for Mental Health, University of Birmingham, Birmingham, UK; Institute of Mental Health, University of Nottingham, Nottingham, UK; The Barberry, Birmingham, UK; Mental Health and Wellbeing, University of Warwick, Coventry, UK; Comprehensive Trials Unit, University College London, London, UK; School of Psychology, University of Sussex, Brighton, UK; Wolfson College, University of Cambridge, Cambridge, UK; Department of Health and Social Care, University of Chester, Chester, UK; Institute of Psychiatry, King’s College London, London, UK; Institute of Psychiatry, King’s College London, London, UK; Division of Psychology and Mental Health, University of Manchester, Manchester, UK; Institute of Population Health Sciences, University of Liverpool, Liverpool, UK; School of Computing Science, University of Glasgow, Glasgow, UK; Institute of Infection, Immunity and Inflammation, University of Glasgow, Birmingham, UK; Division of Psychology and Mental Health, University of Manchester, Manchester, UK; Greater Manchester Mental Health Foundation Trust, Prestwich, UK; Manchester Academic Health Sciences Centre, Manchester, UK; Mental Health and Wellbeing, University of Warwick, Coventry, UK; Institute for Mental Health, University of Birmingham, Birmingham, UK

**Keywords:** psychotic disorders, early intervention, logistic regression, prognosis, precision medicine, schizophrenia

## Abstract

Psychosis is a major mental illness with first onset in young adults. The prognosis is poor in around half of the people affected, and difficult to predict. The few tools available to predict prognosis have major weaknesses which limit their use in clinical practice. We aimed to develop and validate a risk prediction model of symptom nonremission in first-episode psychosis. Our development cohort consisted of 1027 patients with first-episode psychosis recruited between 2005 and 2010 from 14 early intervention services across the National Health Service in England. Our validation cohort consisted of 399 patients with first-episode psychosis recruited between 2006 and 2009 from a further 11 English early intervention services. The one-year nonremission rate was 52% and 54% in the development and validation cohorts, respectively. Multivariable logistic regression was used to develop a risk prediction model for nonremission, which was externally validated. The prediction model showed good discrimination C-statistic of 0.73 (0.64, 0.81) and adequate calibration with intercept alpha of –0.014 (–0.34, 0.31) and slope beta of 0.85 (0.42, 1.27). Our model improved the net-benefit by 16% at a risk threshold of 50% compared to the strategy of treating all, equivalent to 16 more detected nonremitted first-episode psychosis individuals per 100 without incorrectly classifying remitted cases. Once prospectively validated, our first episode psychosis prediction model could help identify patients at increased risk of nonremission at initial clinical contact.

## Introduction

Psychotic disorders, including schizophrenia, are among the 20 leading causes of disability worldwide in 2017. People with psychosis have heterogeneous outcomes with more than 40% not achieving symptomatic remission.^1^ Symptom remission after the first episode of psychosis (FEP) is associated with long-term functional outcome.^2^ The main modifiable reasons for nonremission include treatment resistance,^3^ medication nonadherence,^4^ and comorbid substance misuse.^5^ Although there are effective interventions to ameliorate the reasons for nonremission, there is often a delay in providing these interventions. For people with treatment-resistant psychosis, delays of around 4 years in initiating effective interventions have been reported—for example, clozapine for treatment resistance.^6^ Delay is associated with poorer outcomes. Clinicians have identified the difficulty of early identification of patients likely to become treatment-resistant as a barrier preventing the initiation of effective phase-specific treatments like clozapine at the optimal time.^7^

Early identification of individuals with a higher risk of nonremission at initial clinical contact may facilitate personalized interventions, reduce time to their initiation and improve utilization of resources. Although there have been recent attempts to develop models to predict the individual risk of poor outcome in FEP,^8–10^ these are affected by suboptimal study design and reporting, lack of external validation,^8^ small sample sizes,^9^ and no measures of calibration or clinical utility.^8–10^ This study aimed to develop and externally validate a new prediction model to predict the individual risk of nonremission at one year for individuals with first-episode psychosis.

## Methods

### Data Sources and Study Population

We used data from the National Evaluation of Development of Early intervention Network study (NEDEN) for model development and internal validation. We used data from the Outlook study for external validation. Written informed consent was obtained from all participants. Both studies had NHS Research Ethics Committee approval.

### Development Cohort

NEDEN is a longitudinal naturalistic study of 1027 patients aged 14–35 with FEP recruited from 14 early intervention services across the National Health Service (NHS) in England (2005–2010); the methods and baseline characteristics have been outlined previously.^11^ An analysis of the potential of prediction modeling in FEP using this dataset has been published. We conducted a reanalysis to address methodological issues with our previous analysis^10^ (including the lack of any measures of calibration or clinical utility) and to take advantage of an external validation dataset that was similar to the development dataset (in terms of patients, geography, and clinical service they were drawn from) allowing for better assessment of generalizability. Models in the previous analysis did not inform the present study.

### Validation Cohort

The Outlook study (which was part of the PsyGrid study) is a longitudinal naturalistic study of 399 patients recruited from a further 11 NHS England early intervention services, throughout April 2006–February 2009.^12^ Inclusion criteria: age 16–35, International Classification of Diseases 10^th^ Revision (ICD-10) diagnosis of schizophrenia, schizoaffective disorder, delusional disorder, mania or severe depression with psychosis, acute and transient psychoses, drug-induced psychoses and psychosis not otherwise specified; those with organic brain disorders were excluded.

In both cohorts, participants were recruited as soon after the first contact with the early intervention services as possible. Baseline assessment occurred as soon as a referral was received by a participating service, regardless of whether the potential participant was in the hospital or the community.

### Outcome Measure

Our outcome measure was symptom nonremission at one year. Nonremission was defined as failing to meet the Remission in Schizophrenia Working Group criteria using the Positive And Negative Syndrome Scale (PANSS) at 6 and 12 months, a reliable and valid scale in clinical and research settings. The Remission in Schizophrenia Working Group defined remission as scores of less than or equal to 3 in PANSS items P1 Delusions, P2 Conceptual Disorganization, P3 Hallucinatory Behavior, N1 Blunted Affect, N4 Apathetic Social Withdrawal, N6 Lack of Spontaneity and G9 Unusual Thought Content, present for a period of at least 6 months.^13^

### Candidate Predictors

In both cohorts, psychologists not directly involved in clinical care trained in the use of the rating scales assessed participants at baseline, 6- and 12-month follow-up. Both studies collected candidate predictors based on existing literature and expert knowledge using standardized assessment instruments. These included sociodemographic and clinical variables, the Premorbid Adjustment Scale, PANSS, Young Mania Rating Scale, Birchwood Insight Scale, Calgary Depression Scale for Schizophrenia, Global Assessment of Functioning, and EQ-5D. In addition, participant UK postcode outward code was mapped to primary care trust (PCT). Summary PCT level UK Government Index of Multiple Deprivation (IMD) data (collected between 2001 and 2005, released 2007) was then linked to each patient.

Fourteen predictors were chosen based on previous research and consensus between 5 psychiatrists working in the field of Early intervention in Psychosis. The list of predictors is provided in table 1. As outlined above, similar research involving feature selection was performed using the NEDEN dataset.^13^ This did not influence the choice of predictors for the present study.

**Table 1.** The final logistic regression nonremission prediction model specification. We provide mean and standard deviation values to allow the transformation of the predictor variables to Z-scores for their use in the model.

Variable	Values to transform to Z-score	Unadjusted Final Model		Adjusted Final Model (Shrinkage Factor = 0.84)	
	Mean (SD)	β Coefficient (95% CI)	Odds Ratio (95% CI)	β Coefficient	Odds Ratio
Intercept		0.022 (-0.334, 0.379)		0.029	
Male Sex (1 or 0)	N/A	0.259 (-0.129, 0.646)	1.295 (0.879, 1.908)	0.217	1.242
Age at Study Entry	22.51 (4.887)	-0.037 (-0.210, 0.137)	0.964 (0.810, 1.147)	-0.031	0.970
Past Drug Use (1 or 0)	N/A	-0.101 (-0.478, 0.277)	0.904 (0.620, 1.319)	-0.084	0.919
DUP (days)	307.5 (632.3)	0.546 (0.255, 0.838)	1.727 (1.291, 2.311)	0.460	1.581
PAS Highest Functioning Achieved	1.745 (1.446)	0.427 (0.241, 0.613)	1.533 (1.273, 1.847)	0.358	1.431
PANSS P1 Delusions	2.828 (1.683)	0.060 (-0.166, 0.287)	1.062 (0.847, 1.332)	0.051	1.052
PANSS P2 Conceptual Disorganization	1.945 (1.254)	-0.359 (-0.568, -0.151)	0.698 (0.567, 0.860)	-0.301	0.740
PANSS P3 Hallucinatory Behavior	2.931 (1.686)	0.543 (0.334, 0.753)	1.722 (1.396, 2.123)	0.455	1.577
PANSS N4 Passive Social Withdrawal	2.68 (1.576)	0.346 (0.146, 0.545)	1.413 (1.157, 1.725)	0.290	1.336
PANSS G6 Depression	3.229 (1.681)	-0.198 (-0.398, 0.002)	0.820 (0.672, 1.002)	-0.166	0.847
Insight Scale – Nervous or Mental Illness	1.288 (0.7951)	-0.075 (-0.263, 0.114)	0.928 (0.768, 1.121)	-0.062	0.940
GAF Symptoms	51.48 (16.72)	-0.272 (-0.540, -0.005)	0.762 (0.583, 0.995)	-0.228	0.780
GAF Disability	53.27 (15.58)	-0.019 (-0.267, 0.229)	0.981 (0.765, 1.257)	-0.016	0.984
Average Deprivation Score in Patient’s PCT	27.27 (12.22)	0.221 (0.029, 0.414)	1.248 (1.029, 1.513)	0.185	1.204

DUP, duration of untreated psychosis; PAS, premorbid adjustment scale; PANSS, Positive and Negative Syndrome Scale; GAF, Global Assessment of Functioning; PCT, Primary Care Trust.

### Sample Size Calculation

Using Riley et al’s^14^ criteria for multivariable prediction model development for binary outcomes, the minimum sample size required given a 50% prevalence of nonremission with 14 predictor parameters (meeting the assumptions of global shrinkage factor of ≥0.90, an absolute difference of ≤0.05 between apparent and adjusted R-squared, and a 0.05 margin of error in the estimation of intercept) is 431 with 216 nonremitters (Events per Predictor Parameters [EPP] = 15). Our development cohort included 673 FEP individuals with 353 individuals meeting criteria for nonremission at one year. This provides an EPP of 25, which is above requirements. Further, the number of nonremission events in both the development and validation cohort was >100, which is in line with suggested criteria.^15^ The number of nonevents in the validation cohort was 88, just below suggested criteria.

### Missing Data

Missing data were multiply imputed (*m* = 10) by chained equations using all predictors, auxiliary variables, and outcomes based on the assumption that data was missing at random. Imputed outcome data were then deleted.^16^ It is proposed that this strategy leads to more efficient estimates than an ordinary multiple imputation strategy while also protecting the estimates from problematic imputations in the outcome variable.^15^ This multiple imputation strategy was carried out separately for the development and validation datasets. Ordinal variables were treated as continuous and binary variables were dummy coded. All predictor variables were standardized prior to model construction.

### Statistical Analysis for Model Development

We followed the TRIPOD (Transparent Reporting of a multivariable model for Individual Prognosis Or Diagnosis) guidance for development and reporting of multivariable prediction models.^17^

### Model Development, Internal, and External Validation

A logistic regression model was fitted by maximum likelihood estimation on the 14 chosen predictors. Internal validation performance was assessed by ten-fold cross-validation repeated 5 times on the 10 imputed datasets. The model performance was considered using discrimination and calibration measures. Discrimination, or the ability of our model to distinguish a patient with the outcome (nonremission) from a patient without (remission), was assessed via the C-statistic (with 95% CIs were established via U-statistic theory and permutation testing to confirm significance). Calibration is the level of agreement between the observed outcomes and the model’s predictions. Two measures of model calibration were calculated: calibration-in-the-large (alpha) which is the intercept on the calibration plot and compares mean observed to mean predicted, and, the calibration slope (beta) which relates to the shrinkage of the regression coefficients. A perfectly calibrated model would show an ideal line with intercept alpha of 0 and a slope beta of 1. For internal validation, only the slope beta is of value and corresponds to the shrinkage factor or measure of overfitting.^18^ This uniform shrinkage factor was applied to the final logistic regression model and the intercept was re-estimated prior to external validation on the Outlook dataset.

### Clinical Utility

Clinical utility was assessed in the external validation cohort, in addition to discrimination and calibration. We assessed the clinical usefulness of using a treatment strategy based on the prediction model compared with treating all, treating none, or treating based on the duration of untreated psychosis (DUP) alone (DUP is the most researched and consistent predictor of poor outcome in FEP). Hereto, a decision curve analysis was performed.^19^ Clinical usefulness is considered in terms of net-benefit (the treatment threshold weighted sum of true- minus false-positive classifications for each strategy) plotted against an entire range of treatment thresholds. A treatment threshold is defined as the point where the likelihood of benefit, in our case, reduced rates of nonremission, exactly balances the likelihood of harm. Treatment thresholds vary between individual clinicians and patients depending on their context-specific weighting of relative harms and benefits.


$$\begin{array}{l} Net\;\;Benef\;\;\;it=\;\frac{True\;Positives}{N}\;-\;\frac{False\;Positives}{N}\;x\;\frac{Threshold\;Probabilty}{1-Threshold\;Probability} \end{array}$$

*N* = total sample size.

The intervention (“treatment”) proposed on the prediction of a high risk of nonremission is “enhanced monitoring” over routine care leading to early identification and intervention for treatment resistance, substance misuse, or nonconcordance. To use a prediction model for such treatment decisions, we require to specify a probability threshold above which we would consider the treatment.

We consulted NHS early-intervention specialists (8 NHS Consultant Psychiatrists) to ascertain the probability threshold at which they would consider treatment. The range of thresholds varied between 40% and 60% That is; they would adopt an assertive monitoring and intervention approach when an individual’s probability of nonremission is above 40%–60% to balance the likelihood of benefits versus the harms/costs (in this case, the benefit of reduced rates of nonremission against the probability of harm via intrusive monitoring, side-effects, and increased costs).

Net-benefit is calculated across the range of threshold probabilities of the outcome (nonremission) at which further intervention would be warranted. Net-benefit differs from other performance metrics such as discrimination and calibration because it incorporates the consequences of the decisions made based on a model.

**Fig. 1.**
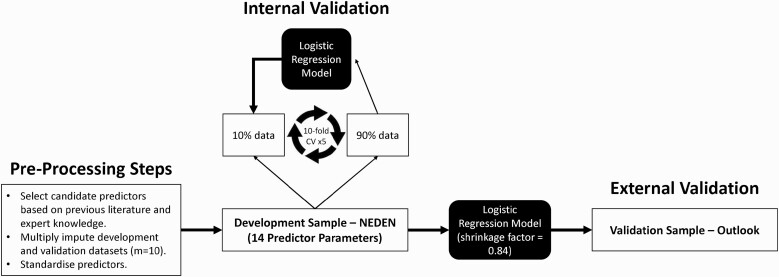
Analysis pipeline.

All analyses were performed using R, CRAN version 4.1.0^20^ (with the “mice,” ^21^ “caret,” ^22^ “pROC,” ^23^ “CalibrationCurves,” and “dca” packages) and code are available online (https://github.com/samleighton87/NEDEN_Outlook_FEP). The analysis pipeline is provided in figure 1.

## Results

### Study Populations

In the NEDEN study, 673 (66%) of 1027 participants had outcome data, of which 353 (52%) met criteria for one-year symptom nonremission. In the Outlook study, 191 (48%) of 399 participants had outcome data, of which 103 (54%) met criteria for nonremission. The baseline characteristics of the development (NEDEN) and validation (Outlook) cohorts are summarized in supplementary table 1.

### Model Development and Internal Validation

The 14 variable logistic regression prediction model is specified in table 1.

At internal validation, the discrimination C-statistic was 0.74 (0.72, 0.76), while the calibration slope beta of 0.84 (0.76, 0.92). This shrinkage factor was applied to the final model coefficients and the intercept was re-estimated.

### External Validation

**Fig. 2.**
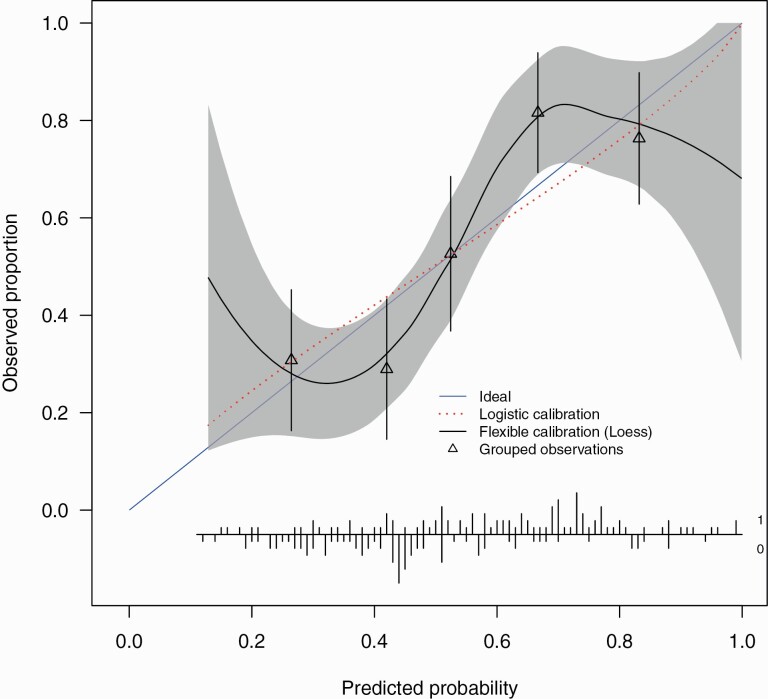
External validation calibration plot. The calibration intercept of -0.014 (-0.34, 0.31) and slope 0.85 (0.42, 1.27). Triangles represent quintiles of subjects grouped by similar predicted risk. The distribution of subjects is indicated with spikes at the bottom of the graph, stratified by endpoint (nonremitters above the x-axis, remitters below the x-axis). Although both sets of confidence intervals overlapped the ideal values, the calibration slope point estimate is smaller than 1 indicating that the predicted risks were too extreme in the sense of overestimating for patients at high risk while underestimating for patients at low risk and is indicative of overfitting of the model. The calibration intercept point estimate was close to ideal suggesting no general over- or underestimation of predicted risks.

At external validation, the model showed fair discrimination with a C-statistic of 0.73 (0.64–0.81). There was a good spread of risk, with good correspondence between observed proportions with psychosis for subjects grouped by similar predicted risk (figure 2).

**Fig. 3.**
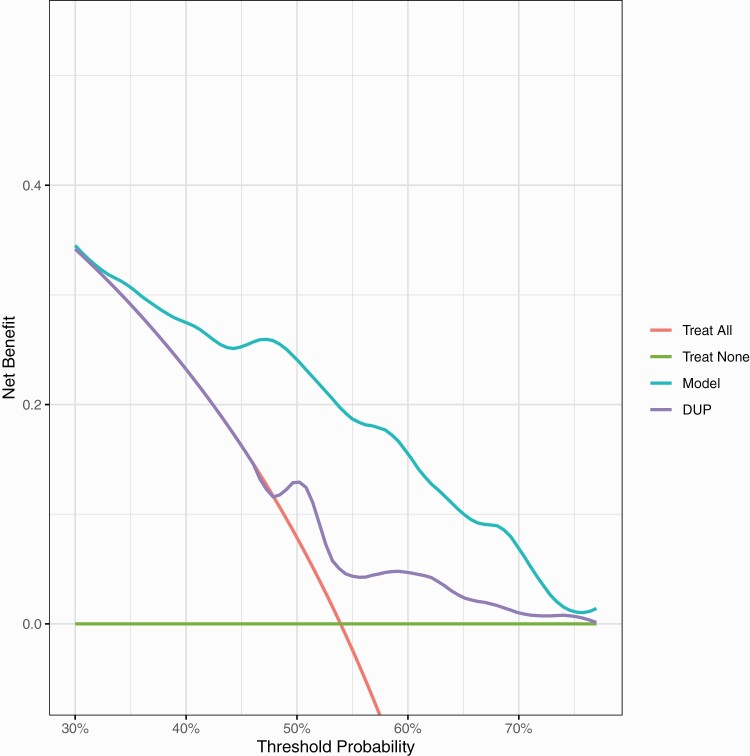
External validation decision curve analysis plot. Net-benefit is the treatment threshold weighted sum of true- minus false-positive classifications for each strategy plotted against an entire range of treatment thresholds. Green line: no patients are treated, net-benefit is zero (no true-positive and no false-positive classifications); red line: all patients are treated; purple and cyan lines: patients are treated if predictions exceed a threshold, with nonremission predictions based on adjusted DUP only, or on our prediction model. Between thresholds of 35% to 70%, treating based on our model is better than treating all, treating none or treating using DUP alone.

For the Outlook external validation, the 54% overall rate of nonremission at one year implies a maximal net-benefit of 54% at a decision threshold for treatment of 0%. Figure 3 shows that between thresholds of 35% to 70% treating based on our model is better than treating all, treating none or treating using DUP alone. At a probability threshold of 50% (midpoint of the range of clinician chosen thresholds), treating based on our model has an increased net-benefit of 16% compared the strategy of treating all, equivalent to 16 more detected nonremitted FEP individuals per 100 FEP individuals without an increase in incorrect classification of remitted FEP individuals as high risk.

## Discussion

We have developed a new risk prediction model based on baseline demographic and clinical variables to predict the risk of nonremission at one year after the onset of first-episode psychosis in a large sample of FEP individuals. The model was validated across services in the development population and externally validated on an independent cohort. The prediction model had fair discrimination and was fairly well-calibrated. The model showed an increase in net benefit.

### Strengths and Weakness of the Study

Our study has some strengths. Both our development and validation cohorts included a representative sample of FEP participants from early intervention services in England, who were prospectively followed up for a year. Both the cohorts were assembled in similar services (early intervention) and periods in England which improves generalizability to patients within these services, though they have potentially changed in the past 10 years resulting from financial austerity measures. The candidate predictors and outcomes were measured using standardized instruments by graduate psychologists who were not directly involved in the care of the participants, which minimized the measurement bias. We used an operationalized, and well-established outcome definition for nonremission, which further minimizes measurement bias. We provided 4 measures of model performance—discrimination, 2 measures of calibration, and decision curve analysis.^18^ Though the baseline measures were meant to be measured on the first presentation to EIS, in practice, there was variation: in NEDEN 32% within 3 weeks of presentation; in Outlook 21% within 3 weeks. The model will apply to patients at least 3 weeks after their presentation to early intervention services.

There are some weaknesses to the study. Ethnicity was not included as a predictor in our model. This is some evidence that treatment resistance may be predicted by ethnicity.^3^ Only around half of the eligible participants consented to participate in the NEDEN study, which may affect the generalizability of our models to the general FEP population. However, those who did not consent were largely similar at baseline to people who did.^11^ Outcome data was not available for 34% of the NEDEN cohort and 52% in the Outlook cohort, which could further limit the validity of the results. As a result, while the number of events in the validation cohort was >100 (103), the number of nonevents was <100 (88). This is slightly less than suggested criteria. The method used for imputation of missing predictors using all the available data including outcome data and deletion of imputed outcome data has the advantage of the predictor imputation benefitting from the full data structure, whilst protecting the regression estimates from often problematic outcome imputations.^15^ This approach has been subject to criticism,^24^ though it is recognized that outcome imputation remains controversial. Further, there were differences in rates of missing data between the development and validation datasets. The outcome was measured only at the 6- and 12-month time points. Study subjects may not have met remission criteria for the entire 6 months in between. The cohorts did not collect biomarkers of illness including inflammatory or neuroimaging data which a previous study in clinical high-risk populations has found to increase prognostic certainty when added to models based on clinical variables.^25^ Another weakness is that we have not accounted for treatment effects, which can lead to suboptimal model performance, albeit only in the presence of strong treatment effects. We assumed that standardized treatment was provided to all participants as they were drawn from early intervention services.

### Comparison With Previous Studies

Three prediction models for outcome in first-episode psychosis have been reported, though they are yet to be used in clinical practice. One study has examined the prediction of social recovery in FEP participants from an RCT,^8^ while the other 2 studies have examined prediction for remission and recovery measures in cohort studies.^9,10^ The discrimination performance for the remission outcome in our study is higher than that reported for models in 2 previous studies (C-statistic of 0.63 on external validation,^9^ and 0.70 and 0.61 on internal and external validation respectively^10^), which could be explained by the smaller sample sizes used in their development and validation,^9^ and the higher number of predictors used for their model development.^10^ Measures of calibration and clinical usefulness have not been provided by the other 2 studies, which adds to the novelty and importance of the current study.

### Implications for Clinicians and Policymakers

The early identification of FEP individuals with higher risk prediction of nonremission may allow for changes to their treatment strategies, leading to improved remission rates. Though suggestions for such a strategy to improve remission rates have been made previously, there have been limited attempts towards a targeted approach to identify FEP individuals at high risk of nonremission.

Health services globally has introduced measures to improve access to services and to ensure that FEP individuals receive evidence-based care. A validated prediction model closely aligns with the policy agenda of early identification of FEP individuals with a high risk of nonremission so that their care can be optimized, and resources targeted according to need.

### Future Research

Prospective validation in additional cohorts from plausibly related settings is required to establish the utility of our model in clinical settings. This will help to compare the model predictions versus clinical intuition and address the issue of treatment effect. Future research also needs to address what biomarkers, such as neuroimaging and immune markers, add to the performance of the model. The model should be validated in a range of clinical settings for its use in services outside England, and in settings that do not have early intervention psychosis services, which may show a need for local updating to improve the accuracy of predictions for specific settings.

## Supplementary Material

sgab041_suppl_Supplementary_Table

## Data Availability

M.B. acts as custodian of the NEDEN dataset and data sharing, and secondary analyses are supported under the auspices of the University of Warwick (Coventry, UK); please contact M.B. for all requests. S.W.L. acts as custodian of the Outlook/PsyGrid dataset and data sharing, and secondary analyses are supported under the auspices of the University of Manchester (Manchester, UK); please contact S.W.L. for all requests.
