# Supplementary material for: Development and Validation of a Nonremission Risk Prediction Model in First-Episode Psychosis: An Analysis of 2 Longitudinal Studies
Source: Schizophr Bull Open. 2021 Aug 31;2(1):sgab041. doi: 10.1093/schizbullopen/sgab041 (PMC8458108; doi:10.1093/schizbullopen/sgab041)
Supplement: sgab041_suppl_Supplementary_Table [file sgab041_suppl_supplementary_table.doc]

**Supplementary Table 1 – Baseline characteristics of the development (NEDEN) and validation (Outlook) cohorts**

| **Baseline**  **Characteristic** | **Development Cohort (NEDEN)**  (n=1027) | | **Validation Cohort (Outlook)**  (n=399) | | **P-Value** |
| --- | --- | --- | --- | --- | --- |
|  | **All** | **With Outcome Data**  (n=673; 66%) | **All** | **With Outcome Data**  (n=191; 48%) |  |
| **Age (Years)**  Mean (SD)  Missing | 22.5 (4.89)  1 (0.1%) | 22.6 (5.01)  1 (0.1%) | 25.6 (7.91)  0 (0%) | 25.0 (7.84)  0 (0%) | ANOVA  *F*(3,2284) = 35.7, *p* =  <0.001* |
| **Sex**  Male  Female  Missing | 709  (69.0%)  318  (31.0%)  0 (0%) | 463  (68.8%)  210  (31.2%)  0 (0%) | 246  (61.7%)  153  (38.3%)  0 (0%) | 118  (61.8%)  73  (38.2%)  0 (0%) | Chi-squared test  χ^2^(3) = 10.4, *p* = 0.015* |
| **In Employment, Education or Training**  Missing | 284  (32.5%)  154 (15.0%) | 190  (33.2%)  100  (14.9%) | 174  (43.6%)  0 (0%) | 85  (44.5%)  0 (0%) | Chi-squared test  χ^2^(3) = 22.6, *p* = <0.001* |
| **Highest Qualification**  None  GCSE/NVQ level 1 or 2  A-level/GNVQ/  BTEC/NVQ level 3  Degree/HND/NVQ level 4 or above  Missing | 245  (24.4%)  399  (39.7%)  262  (26.1%)  98 (9.8%)  23 (2.2%) | 156  (23.7%)  255  (38.8%)  173  (26.3%)  74 (11.2%)  15 (2.2%) | 89  (23.4%)  130  (34.2%)  92  (24.2%)  69 (18.2%)  19 (4.8%) | 40  (21.3%)  67  (35.6%)  46  (24.5%)  35 (18.6%)  3 (1.6%) | Chi-squared test  χ^2^(9) = 26.2, *p* = 0.002* |
| **Adjusted Duration of Untreated Psychosis (Days)**  Mean (SD)  Missing | 308 (633)  16 (1.6%) | 289 (589)  7 (1.0%) | 293 (839)  62 (15.5%) | 342 (1000)  39 (20.4%) | ANOVA  *F*(3, 2162) = 0.291, *p* = 0.832 |
| **Average Deprivation Score in Patient’s Primary Care Trust**  Mean (SD)  Missing | 27.3 (12.2)  6 (0.6%) | 26.6 (11.6)  4 (0.6%) | 30.0 (8.0)  209 (52.4%) | 28.9 (7.9)  108  (56.5%) | ANOVA  *F*(3, 1959) = 5.0, *p* = 0.002* |
| **Positive and Negative Syndrome Scale Total**  Mean (SD)  Missing | 62.7 (18.8)  105 (10.2%) | 64.1 (19.0)  42 (6.2%) | 63.0 (15.5)  67 (16.8%) | 60.9 (14.5)  18 (9.4%) | ANOVA *F*(3, 2054) = 1.64, *p* = 0.178 |

* indicates significance after Bonferroni-Holm correction (n = 7)
